# Reinterpreting published tDCS results in terms of a cranial and cervical nerve co-stimulation mechanism

**DOI:** 10.3389/fnhum.2023.1101490

**Published:** 2023-06-21

**Authors:** Alireza Majdi, Boateng Asamoah, Myles Mc Laughlin

**Affiliations:** ^1^Exp ORL, Department of Neuroscience, Leuven Brain Institute, KU Leuven, Leuven, Belgium; ^2^Leuven Brain Institute, KU Leuven, Leuven, Belgium

**Keywords:** tDCS, transcranial, transcutaneous, working memory, healthy

## Abstract

Transcranial direct current stimulation (tDCS) is a non-invasive neuromodulation method that has been used to alter cognition in hundreds of experiments. During tDCS, a low-amplitude current is delivered *via* scalp electrodes to create a weak electric field in the brain. The weak electric field causes membrane polarization in cortical neurons directly under the scalp electrodes. It is generally assumed that this mechanism causes the observed effects of tDCS on cognition. However, it was recently shown that some tDCS effects are not caused by the electric field in the brain but rather *via* co-stimulation of cranial and cervical nerves in the scalp that also have neuromodulatory effects that can influence cognition. This peripheral nerve co-stimulation mechanism is not controlled for in tDCS experiments that use the standard sham condition. In light of this new evidence, results from previous tDCS experiments could be reinterpreted in terms of a peripheral nerve co-stimulation mechanism. Here, we selected six publications that reported tDCS effects on cognition and attributed the effects to the electric field in the brain directly under the electrode. We then posed the question: given the known neuromodulatory effects of cranial and cervical nerve stimulation, could the reported results also be understood in terms of tDCS peripheral nerve co-stimulation? We present our re-interpretation of these results as a way to stimulate debate within the neuromodulation field and as a food-for-thought for researchers designing new tDCS experiments.

## 1. Introduction

Numerous studies have employed transcranial direct current stimulation (tDCS), a non-invasive neuromodulation technique, to alter cognitive functions (Nikolin et al., 2018; Ke et al., 2019; Karthikeyan et al., 2021). Although research on tDCS applications is growing, surprisingly little is known about the basic neurophysiological mechanisms that underlie the technique (van Boekholdt et al., 2021).

During tDCS, scalp electrodes deliver a low amplitude current, creating a weak electric field in the brain. Directly beneath these electrodes, the weak electric field causes membrane polarization by altering the resting membrane potential in cortical neurons. Anodal tDCS depolarizes the membrane potential and enhances local neural activity while cathodal tDCS hyperpolarizes the membrane potential and suppresses local neural activity (Nitsche et al., 2008). It is generally assumed that this change caused by the electric field in the brain causes the observed effects of tDCS on cognition (Chase et al., 2020). We refer to this as the transcranial mechanism. However, how might large fluctuations in cortical excitability observed in human tDCS research be explained by this weak electric field? Experiments performed on brain slices offer a potential solution to the problem. Recent studies showed that the membrane potential shift at the synapse can be up to four times higher than the shift at the soma (Romero Lauro et al., 2014; Chakraborty et al., 2018), meaning that the level of axon terminal polarization had already reached a point where it was capable of causing significant changes in the dynamics of action potentials (Kronberg et al., 2017).

On the other hand, there is a substantial and ongoing disagreement over the notion that electrical stimulation of the scalp directly modifies brain activity in a regionally limited manner (Vöröslakos et al., 2018; Asamoah et al., 2019). According to recent studies, up to 75% of the administered current may not reach the brain (Lefaucheur et al., 2017). In that light, recent research revealed that some of the effects of tDCS are caused by co-stimulation of the cranial and cervical nerves in the scalp which can also modulate cognition (Vanneste et al., 2020; Luckey et al., 2022). We refer to this as the transcutaneous route.

Evidence suggests that stimulation of peripheral nerves such as the vagus, greater occipital, and trigeminal nerves can activate the locus coeruleus-noradrenergic (LC-NA) system (McIntyre et al., 2012; Vanneste et al., 2020). This system has long been recognized for its role in processing salient inputs and cognitive flexibility. More recent studies showed that LC activity promotes spatial and perceptual learning in the hippocampus (Kaufman et al., 2020).

Nevertheless, in tDCS investigations that employ the standard sham condition; where the current is switched on for only a few seconds before being ramped down to zero, this transcutaneous route is not taken into account. Even studies that sought an ideal sham condition for tDCS experiments did not consider the transcutaneous route (Palm et al., 2013; Garnett and den Ouden, 2015).

The rationale for this study is to provide a novel perspective on the mechanisms behind tDCS by attempting to reinterpret published tDCS results in terms of somewhat overlooked cranial and cervical nerve co-stimulation mechanism. Previous research has primarily focused on the direct modulation of cortical activity through changes in the neuronal membrane potential, but there is evidence to suggest that tDCS may also activate cranial and cervical nerves, which in turn could have an impact on cortical activity.

By doing so, the study hopes to shed light on the potential mechanisms behind tDCS and provide a foundation for future studies in this area. Also, this study aims to stimulate debate within the non-invasive brain stimulation community and to encourage researchers planning new tDCS studies to carefully consider these issues when designing their experiments.

## 2. Study selection

Using the keywords “transcranial direct current stimulation,” “working memory,” and “healthy,” we electronically searched Medline *via* PubMed for studies that evaluated the effects of tDCS on working memory (WM) in healthy volunteers. The search was limited to randomized and non-randomized studies published in English. Publications that included non-healthy subjects were excluded from this study. Also, citations that assessed the effects of cognitive training plus tDCS on WM were not included. These search criteria yielded 41 publications. Further studies were found by a manual search of the references of 2 meta-analyses (Mancuso et al., 2016; Medina and Cason, 2017) in the field. Accordingly, six publications were considered eligible for further investigation; as only these publications provided a mechanistic view of their findings.

## 3. Summary of published tDCS studies

### 3.1. Study 1: “Anodal tDCS augments and preserves working memory beyond time-on-task deficits”

Karthikeyan et al. (2021) investigated the effects of the left dorsolateral prefrontal cortex (DLPFC) anodal tDCS on performance during a fatiguing visuospatial WM test. 32 healthy participants underwent anodal, control, and sham tDCS on different days in a repeated measures approach. They completed an hour-long two-back task that assessed working memory, with stimulation intensity at 1 mA, onset at the 20th min, and duration set, for 10 min. This study discovered that short-duration anodal tDCS at 1 mA enhanced and preserved WM capacity both in males and females. This effect persisted after the stimulation interval and was unique during performance assessments. Besides, anodal tDCS was an effective countermeasure to time-on-task (the length of time spent actively involved in a task) deficiencies during a visuospatial two-back task (Karthikeyan et al., 2021).

### 3.2. Study 2: “The effects of stress and transcranial direct current stimulation (tDCS) on working memory: a randomized controlled trial”

In a randomized controlled trial Ankri et al. (2020) assessed the effects of tDCS on WM in healthy individuals. 69 female participants were randomly assigned to four experimental conditions: stimulation (DLPFC tDCS vs. sham stimulation) and stress modification. The current with an intensity of 2 mA was applied for 20 min with a fade-in/fade-out ramp of 30 s. The goal was to investigate the interaction between social stress and tDCS on WM performance. The n-back task was used to measure participants’ attention and WM performance. They showed that active tDCS may improve WM function under no-stress situations while impairing it under stressful conditions. Authors argued that perhaps increasing executive attention and enabling individuals to perform at their best levels in the WM requires either creating mild stress or using DLPFC tDCS. On the contrary, when confronted with extreme stress or, as in the current study, when mild stress and tDCS are coupled, individuals may experience overload, which would impede task execution. This theory postulates that right DLPFC tDCS and stress both alter the activity of overlapping PFC neural networks and that their combined effects are harmful (Ankri et al., 2020).

### 3.3. Study 3: “Transcranial direct current stimulation and working memory: comparison of effect on learning shapes and English letters”

In this double-blinded, randomized, crossover, sham-controlled study Ramaraju et al. (2020) recruited twenty healthy volunteers who were randomized to either active or sham anodal tDCS to their left DLPFC. During stimulation current was ramped up to 1.5 mA over 8 s, followed by continuous stimulation at 1.5 mA. A minimum of 1 week passed between the sham and actual anodal tDCS sessions. To assess recall accuracy participants were subjected to two 2-back-tasks. First, they were asked to recall English letters (A-L), which had been presented in random order. In the second they were presented with 12 geometric shapes. The combination of memory tasks and anodal tDCS had a significant impact on WM. Where the geometric shapes approach had a large effect size, the letters-based approach showed a smaller effect size. The authors thought this might result from differences in the spatial structures utilized for each stimulus (Ramaraju et al., 2020).

### 3.4. Study 4: “Testing the limits: investigating the effect of tDCS dose on working memory enhancement in healthy controls”

Hoy et al. (2013) examined how changing the current (or “dose”) of tDCS affected WM performance in 18 healthy controls on both a behavioral and neurophysiological level. Over the course of 3 weeks, single sessions of 1 mA, 2 mA, and sham anodal tDCS to the left PFC were conducted. At 0, 20, and 40 min after stimulation, participants completed a WM task while EEG was recorded. The results showed that 1 mA had the greatest impact on the WM task. These results were unexpected given that tDCS dosage effects on cognitive improvement have been demonstrated in patient groups in the past. This meant that active anodal left PFC tDCS could improve WM performance in some ways, including speeding up responses on the 2-back task, greater effects were not shown at higher doses in healthy individuals. tDCS showed no effect on the EEG response for correct answers on the 3-back task. Regardless of the stimulation setting, theta event-related synchronization (ERS) declined with time and alpha event-related desynchronization (ERD) rose over time (Hoy et al., 2013).

### 3.5. Study 5: “Unilateral prefrontal direct current stimulation effects are modulated by working memory load and gender”

In a study focused on executive attention, Meiron and Lavidor (2013) used a modified verbal n-back task. They looked at implicit learning in a post-stimulation WM task and “online (during WM activity)” WM performance while 41 healthy participants underwent tDCS. The three conditions were: (1) active left DLPFC anodal stimulation; (2) active right DLPFC anodal stimulation; (3) sham left DLPFC anodal stimulation. 2 mA current was delivered under active tDCS settings for 15 min, with a 30 s fade in/fade out ramp. They showed that men performed better under left DLPFC stimulation whereas women performed better under right DLPFC stimulation, according to significant lateralized “online” stimulation effects that were only observed in the condition with the highest WM load. Performance under high WM loads during left DLPFC stimulation was highly correlated with post-stimulation recall (Meiron and Lavidor, 2013).

### 3.6. Study 6: “Prefrontal direct current stimulation modulates resting EEG and event-related potentials in healthy subjects: a standardized low-resolution tomography (sLORETA) study”

In a cross-over double-blind, placebo-controlled experiment Keeser et al. (2011) used spectral power analysis and sLORETA to assess the distribution of neuronal electrical activity changes following anodal tDCS of the left DLPFC and cathodal tDCS of the right supraorbital area. They subjected ten healthy volunteers to both active and sham tDCS on different days. Anodal tDCS was used over the left DLPFC for 20 min at 2 mA intensity, with the cathode placed over the opposite supraorbital area. Following tDCS, EEG was captured during an eyes-closed resting state and an n-back task where numbers were presented. In the active tDCS condition, statistical non-parametric mapping revealed decreased left frontal delta activity. The next n-back task demonstrated that prefrontal tDCS had a favorable effect on error rate, accuracy, and response time. This was followed by an increase in the P2 and P3 amplitudes of the event-related potentials (ERP). Also, sLORETA source localization for the time range 250–450 ms (post-event) revealed increased activity in the left parahippocampal gyrus. The authors concluded that in addition to enhancing working memory function, anodal tDCS of the left DLPFC and/or cathodal tDCS of the opposite supraorbital region may modulate local electrical activity in the prefrontal and anterior cingulate cortex (Keeser et al., 2011).

## 4. Studies’ opinion of tDCS mechanism of action

What are the tDCS mechanisms of action? This issue has not yet been resolved. Currently, the transcranial mechanism of tDCS is thought to be the main pathway of action (Lefaucheur and Wendling, 2019; Chase et al., 2020). When tDCS is administered at 1–2 mA, the peak electric field value is at most 1 V/m (0.2–2 V/m or 0.05–0.5 A/m^2^ current density), which causes a shift of around 0.2 mV in membrane polarization (Jackson et al., 2016; Modolo et al., 2018). In that light, the cited studies (studies 1 through 6 under Section “3. Summary of published tDCS studies”) have postulated that the effects may be because of a weak direct current that modifies the resting potential of cortical neurons. It has been proposed that tDCS alters neurons’ responsiveness (e.g., firing rate) to endogenous stimuli or modifies neurons’ spontaneous firing rate (Meiron and Lavidor, 2013; Ankri et al., 2020). However, each of these studies has proposed a distinct major downstream influence mediating observed effects in humans, described in the following four Sections “4.1. Modulation of cortical networks activity, 4.2. Modulation of cortical oscillatory activity, 4.3. Long-term potentiation, and 4.4. GABA release.”

### 4.1. Modulation of cortical networks activity

Karthikeyan et al. attributed the observed effects to the direct activation of different cortical networks, such as the DLPFC, which underlies processes involved in WM (Rowe et al., 2000). This suggested the non-focal properties of tDCS which were dependent on the electrode size, current density as well as intensity (Karthikeyan et al., 2021). In the Meiron and Lavidor (2013) study, authors suggested a similar mechanism. They proposed excitability shifts within the frontoparietal network by the superior longitudinal fasciculus (SLF). This was concluded when their specific unilateral tDCS montage, which directs a constant current from the superior parietal cortex (cathodal over Cz) to the DLPFC, was used. Because of this, they stated that the tDCS montage they used may have stimulated the connection between the perceptual and executive components of the n-back task, increasing DLPFC control over information under high WM load (Meiron and Lavidor, 2013).

### 4.2. Modulation of cortical oscillatory activity

Oscillations are ubiquitous throughout the brain and are believed to underpin a wide range of brain functions. Particularly, it is thought that theta (4–8 Hz) and alpha (8–13 Hz) oscillations support cognitive activities (Klimesch, 1999). Both enhanced theta ERS and increased alpha ERD have been linked to WM functioning, with stronger effects as WM demands rise (Pesonen et al., 2007). Hoy et al. (2013) suggested the regulation of cortical oscillatory activity as one possible underlying mechanism. Accordingly, the authors suggested that anodal PFC tDCS modifies cortical oscillatory activity; more specifically theta and alpha powers (Zaehle et al., 2011). Given the role of these oscillatory bands in cognition, this could explain the improved performance of participants in the WM task. In that light, they concluded that tDCS causes improved performance through the manipulation of oscillations.

### 4.3. Long-term potentiation

Ramaraju et al. (2020) argued that during anodal tDCS the cerebral cortex undergoes polarity-dependent alterations. In this study, long-term potentiation (LTP) was thought to be a natural inducer of polarity-dependent changes (Ramaraju et al., 2020). It was shown that anodal tDCS causes NMDA receptor-dependent long-lasting synaptic potentiation in mouse M1 slice (Rroji et al., 2015). Briefly, numerous neurotransmitters phosphorylate cAMP-responsive element binding protein (CREB) and activate genes in the nucleus of neurons by activating or inhibiting transduction cascades that are coupled to G-proteins or ion channels. The creation of numerous proteins, including neurotransmitter synthases, receptors, ion channels, and intracellular signal proteins, is facilitated by these signal transduction cascades. The capacity of tDCS to generate LTP may be explained by the facilitative activities of these proteins that control the effectiveness of neurotransmissions in the cerebral cortex circuit (Yamada and Sumiyoshi, 2021). On the other hand, using cathodal tDCS did not affect LTP, which implies that tDCS-dependent LTP induction is polarity specific (Ranieri et al., 2012).

### 4.4. GABA release

In Keeser et al. (2011) study, magnetic resonance spectroscopy (MRS) measurements of alterations in GABA levels following anodal tDCS indicated that anodal excitatory effects also influence GABAergic inhibition in addition to NMDA-receptor dependence.

## 5. Reinterpretation of tDCS findings in terms of a cranial and cervical nerve co-stimulation mechanism

In this study, the reviewed six papers were chosen because they assessed the effects of tDCS on cognitive function in healthy individuals along with providing an explanation on the possible mechanisms that were involved in the observed effects. Other studies were excluded as they either did not assess the effects of tDCS on cognitive processes, they were performed on non-healthy individuals, or did not give any explanations on the possible mechanisms that might be involved in their observed effects. So, it was not possible for us to judge their view in this regard.

It is certainly possible that tDCS works through direct transcranial modulation of target areas in the brain as proposed in the six studies reviewed (Lefaucheur and Wendling, 2019). However, during conventional tDCS in human subjects cranial and cervical nerves may fire action potentials. This causes the well-known scalp tingling sensation typically experienced by tDCS subjects (Kessler et al., 2012). The trigeminal nerve (fifth cranial nerve) can be stimulated using the anterior tDCS montage which could then alter brain activity. This nerve is subjected to electric fields as high as 20 V/m during tDCS (So et al., 2004). As the largest cranial nerve, trigeminal nerve terminals stretch out across the forehead, jaw, and face (Rea, 2014). This nerve’s afferent fibers proceed to the brainstem, where they end up in the trigeminal nuclei (primary sensory nucleus and spinal nucleus) and nucleus of the solitary tract (NTS) (Tyler et al., 2015). The information is then integrated into the reticular formation which is comprised of the noradrenergic locus coeruleus (LC) and other brainstem nuclei. Trigeminal nerve stimulation increases LC activity thereby increasing noradrenaline release (Tyler et al., 2015). Noradrenaline then enhances glutamate-dependent cortical facilitation and long-term potentiation-like plasticity, increases cortical excitability, and influences motor learning and cognition (Kuo et al., 2021).

In tDCS studies what is usually done is to have a sham condition during which no stimulation is performed on the subjects so that the trigeminal nerve is not stimulated and no noradrenaline is released from LC causing any effects on the target areas such as the hippocampus and DLPFC. On the other hand, due to the stimulation of the trigeminal nerve during active conditions, LC is activated, and noradrenaline is released. Noradrenaline is known to play role in some of the observed cognitive impacts on memory and learning-related areas.

The exact mechanism by which tDCS induces the noradrenaline increase remains unclear (i.e., either transcranial or transcutaneous). However, it is interesting to note that noradrenaline plays a significant role in cognition (Kuo et al., 2017). For example, when older animals are evaluated on the delayed alternation task (in which a non-human animal must alternate responses between trials) with distractor signals, systemic α2- adrenergic receptor (AR) agonism increases behavioral performance, which is consistent with the impact of α2-AR vs. α1-AR agonism on preferred direction delay cell firing (Arnsten and Contant, 1992). Further, in the delayed alternation task, noradrenaline deficiency results in minor working memory deficits (Brozoski et al., 1979).

Locus coeruleus activation also improves 5-HT (5-hydroxytryptamine) neurons’ activity *via* its monosynaptic excitatory inputs to the dorsal raphe nucleus (DRN) (Manta et al., 2009). This activation, however, seems not to be immediate and becomes statistically significant around 2 weeks after stimulation and remains high enough up to 90 days of stimulation (Dorr and Debonnel, 2006). It is postulated that the LC persistently stimulates the DRN through the activation of excitatory α1-receptors (Baraban and Aghajanian, 1980). The neurons in DRN then send their projections to several downstream targets that are significant in this context (Fanselow, 2012). For example, the medial prefrontal cortex (mPFC) receives a significant serotonergic projection from the DRN and plays an outstanding role in regulating both cognitive and emotional processes. Both pyramidal cells and interneurons of mPFC express various types of 5-HT receptors, with a notable prevalence of the 5-HT_1A_ and 5-HT_2A_ subtypes (Pompeiano et al., 1992; Zhao et al., 2015). Stimulating the raphe nuclei, including the DRN, with electrical impulses leads to the release of 5-HT in the mPFC and results in the inhibition of most pyramidal neurons located in this area (Puig et al., 2005; Wang et al., 2020). Additionally, 5-HT, acting through 5-HT_1A_ receptors located on fast-spiking GABAergic interneurons, reduces the amplitude of prefrontal gamma rhythms (Puig et al., 2010). Gamma rhythms are known to be associated with wakeful cognitive processes such as attention and memory encoding and retrieval (Montgomery and Buzsáki, 2007). This mechanism, however, might not be relevant to the effects we have observed in the six studies assessed here (which have typically used acute stimulation); as they appear only 2 weeks after stimulation.

## 6. An unresolved question: transcranial, transcutaneous, or both?

There is a wide range of tDCS effects on attention, memory, and learning in both clinical and experimental studies. Although this is currently unclear, a variety of transcranial and transcutaneous pathways may mediate the effects of tDCS. However, it is not yet known if the transcranial and transcutaneous pathways of tDCS differentially affect neuronal properties. The fact that possible contributions from the transcutaneous pathway are neither controlled for nor actively studied is now a significant problem in the tDCS field. A standard sham condition, in which the tDCS is merely turned off, is employed as the control in practically all tDCS experimental designs (Fonteneau et al., 2019). Because the standard sham control terminates both the transcranial and transcutaneous mechanisms, it is impossible to determine which mechanism is responsible for the observed tDCS effects. A better experimental design that distinguishes between transcranial and transcutaneous mechanisms would be beneficial for tDCS research. To this end, we strongly advise the inclusion of a new control condition (Kerstens et al., 2022), a “transcranial route” condition, in all upcoming tDCS studies in which the transcutaneous route of tDCS is blocked, e.g., by application of topical anesthetics to the scalp in humans or transdermal injection of anesthesia in animals (Asamoah et al., 2019). This control is readily implementable and requires little change to the experimental setup as a whole.

## 7. Conclusion

Research in tDCS is expanding, nevertheless remarkably little is understood about the neurophysiological principles behind the technique. The assumption that electric stimulation of the scalp directly alters brain activity and does so in a confined location is subject to significant and ongoing debate (Vanneste et al., 2020; van Boekholdt et al., 2021). Accordingly, it was shown that some of the effects of tDCS are caused by simultaneous stimulation of the cranial and cervical nerves in the scalp (Vanneste et al., 2020). It is currently an issue in tDCS research that potential contributions from the transcutaneous route are neither taken into account nor evaluated. In this work, we propose that at least some of the effects reported in tDCS studies in healthy individuals could be attributed to the stimulation of peripheral nerves such as the trigeminal nerve. In that light, better experimental designs that distinguish between transcranial and transcutaneous effects are needed in tDCS research in the future.

## Data availability statement

The original contributions presented in this study are included in the article/supplementary material, further inquiries can be directed to the corresponding author.

## Author contributions

AM: manuscript writing and drafting. MM and BA: critical revise. All authors contributed to the article and approved the submitted version.
